# Liver transplantation for advanced-stage primary hepatic yolk sac tumor: A case report and literature review

**DOI:** 10.1097/MD.0000000000035821

**Published:** 2023-12-15

**Authors:** Guang-Hua Liu, Ming-Ke Qiu, Yang Wang, Ting-Ting Zhang, Li-Jun Wang, Wen-Bin Guan, Jing-Min Ou, Li-Tian Chen

**Affiliations:** a Department of Interventional and Vascular surgery, Xinhua Hospital Affiliated to Shanghai Jiaotong University School of Medicine, Shanghai, China; b Department of Radiology, Xinhua Hospital Affiliated to Shanghai Jiaotong University School of Medicine, Shanghai, China; c Department of Pathology, Xinhua Hospital Affiliated to Shanghai Jiaotong University School of Medicine, Shanghai, China; d Department of General Surgery, Xinhua Hospital Affiliated to Shanghai Jiaotong University School of Medicine, Shanghai, China.

**Keywords:** primary hepatic yolk sac tumor, metastasis, survival, treatment

## Abstract

**Rationale:**

Primary hepatic yolk sac tumors (YSTs) are rare in adults. Liver resection is an acknowledged treatment modality for primary hepatic YST. Liver transplantation may offer a possible cure for unresectable cases.

**Patient concerns:**

We present a case of a 31-year-old woman with an abdominal mass who had abnormally elevated alpha-fetoprotein (AFP) levels (31,132 ng/mL; normal: 0–7 ng/mL). Contrast-enhanced computed tomography (CT) revealed large tumors located in both lobes of the liver, with arterial enhancement and venous washout. Fluorine-18 fluorodeoxyglucose (^18^F-FDG) positron emission tomography (PET)/CT indicated increased ^18^F-FDG uptake (maximum standardized uptake value, 24.4) in the liver tumors and left middle intra-abdominal nodule.

**Diagnoses:**

The diagnosis was primary hepatic YST with metastasis to the greater omentum.

**Interventions:**

The patient underwent orthotopic liver transplantation and intra-abdominal nodule resection after transarterial chemoembolization (TACE) as a bridge. Intraoperatively, an intra-abdominal nodule was confirmed in the greater omentum. Histopathological examination of the liver tumors revealed Schiller-Duval bodies. The tropomyosin receptor kinase (TRK) inhibitor larotrectinib was administered, followed by four cycles of chemotherapy with bleomycin, etoposide, and cisplatin based on the next-generation sequencing results.

**Outcomes:**

The AFP level decreased to within the normal range. No evidence of tumor collapse was observed during the 34-month follow-up period.

**Lessons:**

This case suggests that multimodal therapy dominated by liver transplantation, including preoperative TACE, postoperative adjuvant chemotherapy, and TRK inhibitors, is an effective treatment modality for unresectable primary hepatic YST.

## 1. Introduction

Yolk sac tumor (YST), also known as endodermal sinus tumor, is a type of malignant germ cell tumor.^[1]^ The majority of YSTs originate from the gonads. Extragonadal YSTs are rare and are commonly located in midline structures such as the mediastinum, retroperitoneum, and sacral region.^[2]^ Primary hepatic YST is an extremely rare manifestation of extragonadal YST, with only 15 adult cases reported to date in the English literature (Table 1).^[3–17]^

**Table 1:** Summary of case reports of primary hepatic yolk sac tumors in adults.

Author (year)	Sex	Age (years)	AFP (ng/mL)	Size (cm)	Number	Location	Treatment	Survival	Status
Natori (1983)^[3]^	F	29	4270	17 × 15 × 10	Single	Right lobe	Repeated hepatectomy + postoperative chemotherapy (actinomycin D, vincristine, and cyclophosphamide)	6.6 mo	Dead
Narita (1988)^[4]^	F	27	28,500	11 × 11 × 9.5	Single	Right lobe	Partial hepatectomy	1 yr	Dead
Villaschi (1991)^[5]^	F	28	500	15 × 15 × 15	Single	Left lobe	Partial hepatectomy	NA	NA
Whelan (1992)^[6]^	F	27	89,000	15 × 15	Single	Right lobe	Preoperative chemotherapy (BEP) + right hemihepatectomy	5+ yr	Alive
Higuchi (1993)^[7]^	F	24	115,500	“Large tumor”	Multiple	Right lobe	TACE	NA	NA
Wong (1998)^[8]^	F	28	14,614	15 × 15 × 6	Single	Right lobe	Partial hepatectomy	1 yr	Alive
Gunawardena (2002)^[9]^	M	37	326	10 D	Single	Left lobe	Partial hepatectomy	NA	NA
Toumi (2004)^[10]^	F	28	54,000	13.7 D	Single	Right lobe	Right hepatectomy + postoperative chemotherapy (BEP)	3 mo	Pulmonary metastases
Lenci (2008)^[11]^	M	64	400	2.8 D	Single	Right lobe	Laser ablation + TACE + liver transplantation	12 + mo	Alive
Hosala (2014)^[12]^	F	20	>71,753	15 × 15 × 12	Single	Right lobe	Right hemicolectomy + postoperative chemotherapy (BEP)	48 + mo	Alive
Reznichenko (2016)^[13]^	F	39	34.32	25 D	Multiple	Both lobes	Right extended hepatectomy	3 + wk	Tumor progression
Evers (2018)^[14]^	F	45	>10,000	11.3 × 9.5 × 7.4	Multiple	Right lobe	Preoperative chemotherapy (BEP) + partial hepatectomy	NA	NA
Prilutskiy (2018)^[15]^	F	56	505.6	13 D	Single	Right lobe	Preoperative chemotherapy (EP) + partial hepatectomy	NA	NA
Vanidassane (2019)^[16]^	M	27	120,000	13.6 × 10.6 × 10.6	Single	Both lobes	Chemotherapy (BEP + paclitaxel, ifosfamide, and cisplatin)	NA	NA
Jindal (2021)^[17]^	F	28	55,897	NA	Multiple	Right lobe	Chemotherapy (BEP)	NA	NA
Liu (2023)*	F	31	31,132	14.4 × 11.8 × 10.2	Multiple	Both lobes + metastasis of greater omentum	Preoperative TACE + liver transplantation and omentectomy + postoperative chemotherapy (BEP) + larotrectinib	34 + mo	Alive

BEP = bleomycin, etoposide, and cisplatin, NA = not available, TACE = transcatheter arterial embolization.

* This study.

Owing to the lack of specific laboratory biomarkers and imaging manifestations, it is difficult to differentiate primary hepatic YST from hepatocellular carcinoma (HCC) and hepatoblastoma. Most primary hepatic YSTs are diagnosed by incidental preoperative biopsy or postoperative histopathological examination, and are characterized by the formation of the Shiller-Duval body, that is, central vessels surrounded by epithelial tumor cells and fibrous tissue. Primary hepatic YST can be successfully treated with liver resection alone^[4,5,8,9,13]^ or liver resection combined with adjuvant chemotherapy.^[3,6,10,12,14,15]^ However, primary hepatic YST are often asymptomatic, and many patients have no opportunity for liver resection due to advanced-stage disease. Selected patients with unresectable primary hepatic YST can benefit from liver transplantation. It has been reported that an adult male with hepatic YST under routine follow-up who was initially suspected of having early HCC underwent liver transplantation after unsuccessful laser ablation.^[11]^ Here, we describe a patient with advanced-stage primary hepatic YST and preoperatively suspected HCC who underwent liver transplantation with concurrent extrahepatic metastasectomy along with a literature review to illustrate the clinicopathological characteristics and treatment protocol of this rare liver tumor. To our knowledge, this is the first report of an initially unresectable hepatic YST in a young female who underwent successful liver transplantation with concurrent extrahepatic metastasectomy to achieve good long-term survival through preoperative transarterial chemoembolization (TACE) and postoperative adjuvant chemotherapy, followed by targeted therapy.

## 2. Case presentation

**Figure 1.**
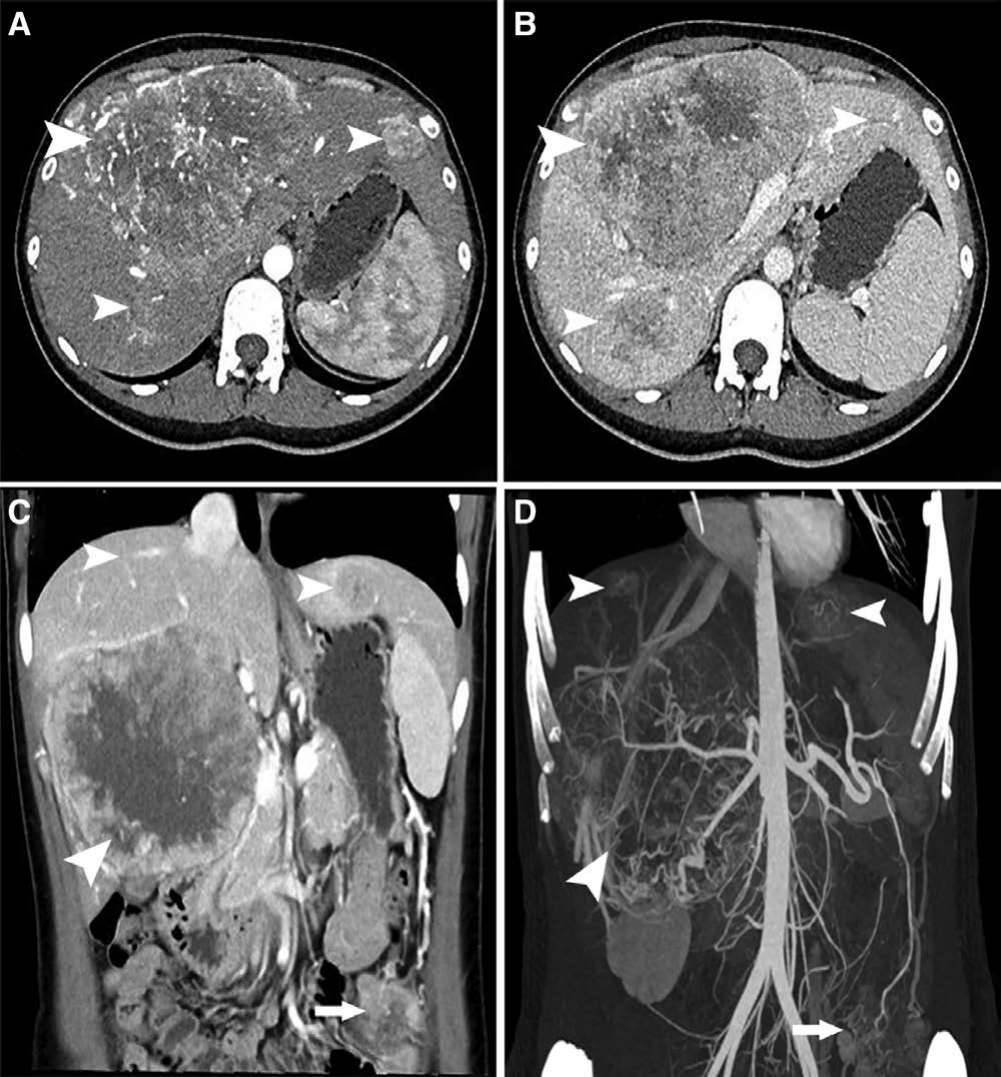
Multiphase contrast-enhanced CT of the liver tumor (arrowheads) and intra-abdominal metastases (arrows). (A) contrast-enhanced CT image in the arterial phase; (B) contrast-enhanced CT image in the venous phase; (C) coronal reformatted CT image; (D) CT vascular reconstruction image. CT = computed tomography.

**Figure 2.**
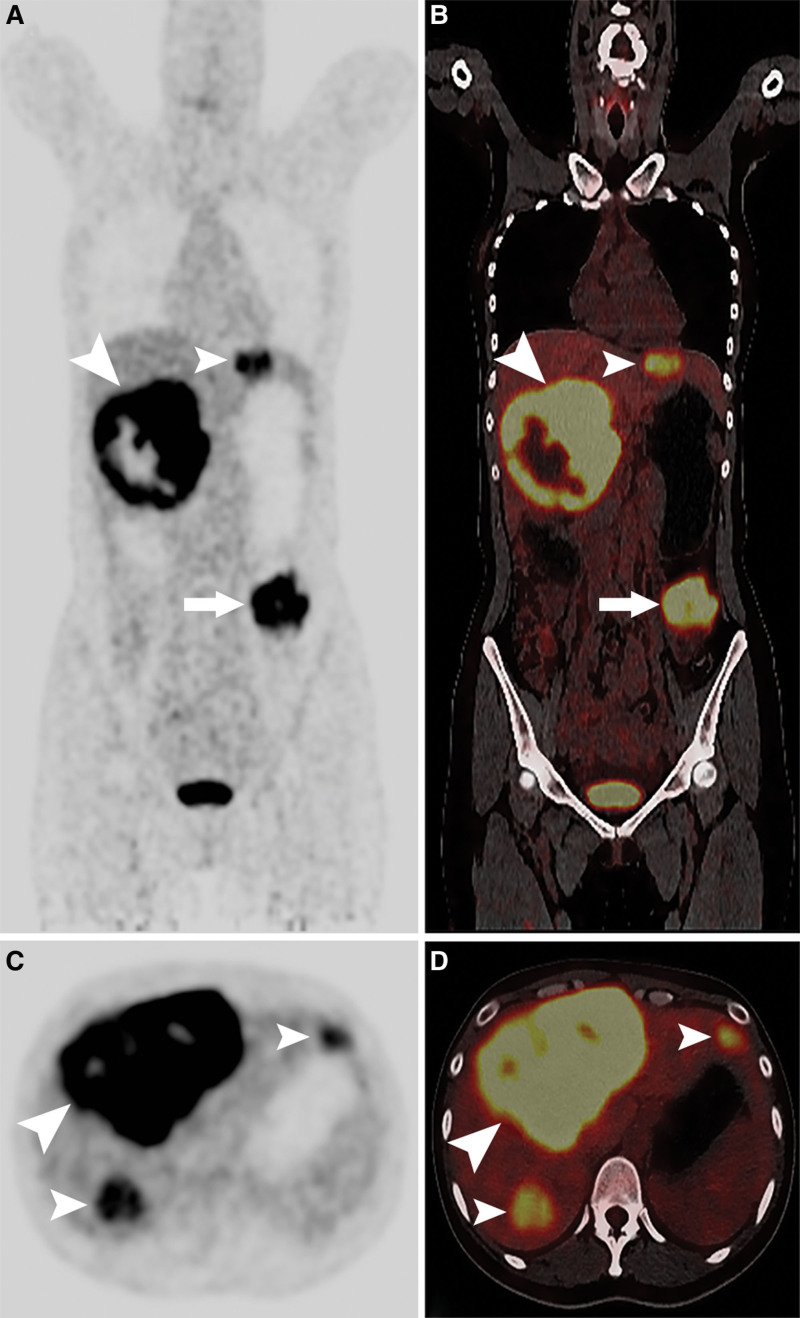
18F-FDG PET/CT images in a case of primary hepatic YST (arrowheads) with intra-abdominal metastasis (arrows). (A) maximum intensity projection 18F-FDG PET; (B) fused PET/CT image; (C) axial PET image; (D) axial fused PET/CT image. ^18^F-FDG = fluorine-18 fluorodeoxyglucose, CT = computed tomography, PET = positron emission tomography, YST = yolk sac tumor.

A 31-year-old woman presented to the general surgery outpatient clinic with abdominal distension and an abdominal mass that had persisted for 1 month. She had no history of alcohol, drug, viral, or metabolic liver diseases. Physical examination revealed a large tough mass in the upper right abdomen. Laboratory examination revealed an alpha-fetoprotein (AFP) level of 31,132 ng/mL (normal range, 0–7 ng/mL), carcinoembryonic antigen and carbohydrate antigen 19-9 levels within the normal ranges, an alanine aminotransferase level of 143 U/L (normal range, 0–75 U/L), an aspartate aminotransferase level of 83 U/L (normal range, 0–38 U/L), and a total bilirubin level of 14.5 µmol/L (normal range, 3.4–20.5 µmol/L), and the hepatitis panel was negative. Multiphase contrast-enhanced computed tomography (CT) revealed large hypodense masses in both lobes of the liver, with heterogeneous peripheral arterial enhancement (Fig. 1A) and venous washout (Fig. 1B). Coronal reformatted CT and three-dimensional vascular reconstruction images indicated multiple intrahepatic masses (Fig. 1C) and intra-abdominal metastasis with a blood supply from the splenic artery (Fig. 1D). The patient was initially diagnosed with HCC with intra-abdominal metastasis. The patient was referred for fluorine-18 fluorodeoxyglucose (^18^F-FDG) positron emission tomography (PET)/computed tomography (CT) restaging. ^18^F-FDG PET/CT revealed multiple hypodense lesions in both lobes of the liver, with clear boundaries, the largest one being located in the right lobe, with dimensions of 14.4 × 11.8 × 10.2 cm, with increased ^18^F-FDG uptake (maximum standardized uptake value, 24.4) in the solid part of the tumors. An area with intense metabolic activity was observed in the left middle abdominal cavity, which strongly suggested intra-abdominal metastasis (Fig. 2A–D).

**Figure 3.**
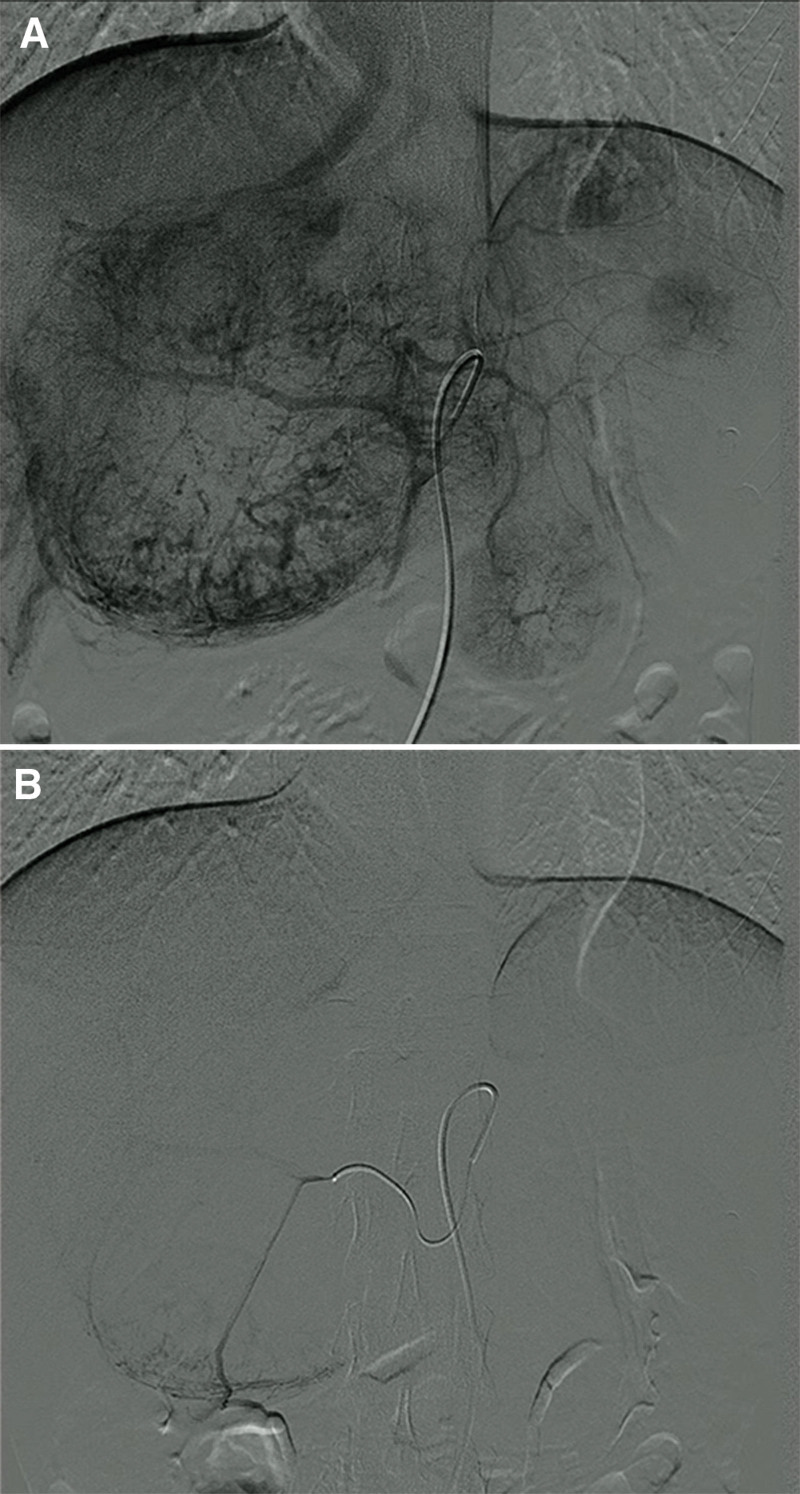
Digital subtraction angiography (DSA) showed hypervascular staining in the liver tumors (A). Transarterial chemoembolization (TACE) was delivered with 1000 mg of fluorouridine followed by a mixed solution of 10 mL lipiodol and 150 mg oxaliplatin under DSA monitoring (B).

**Figure 4.**
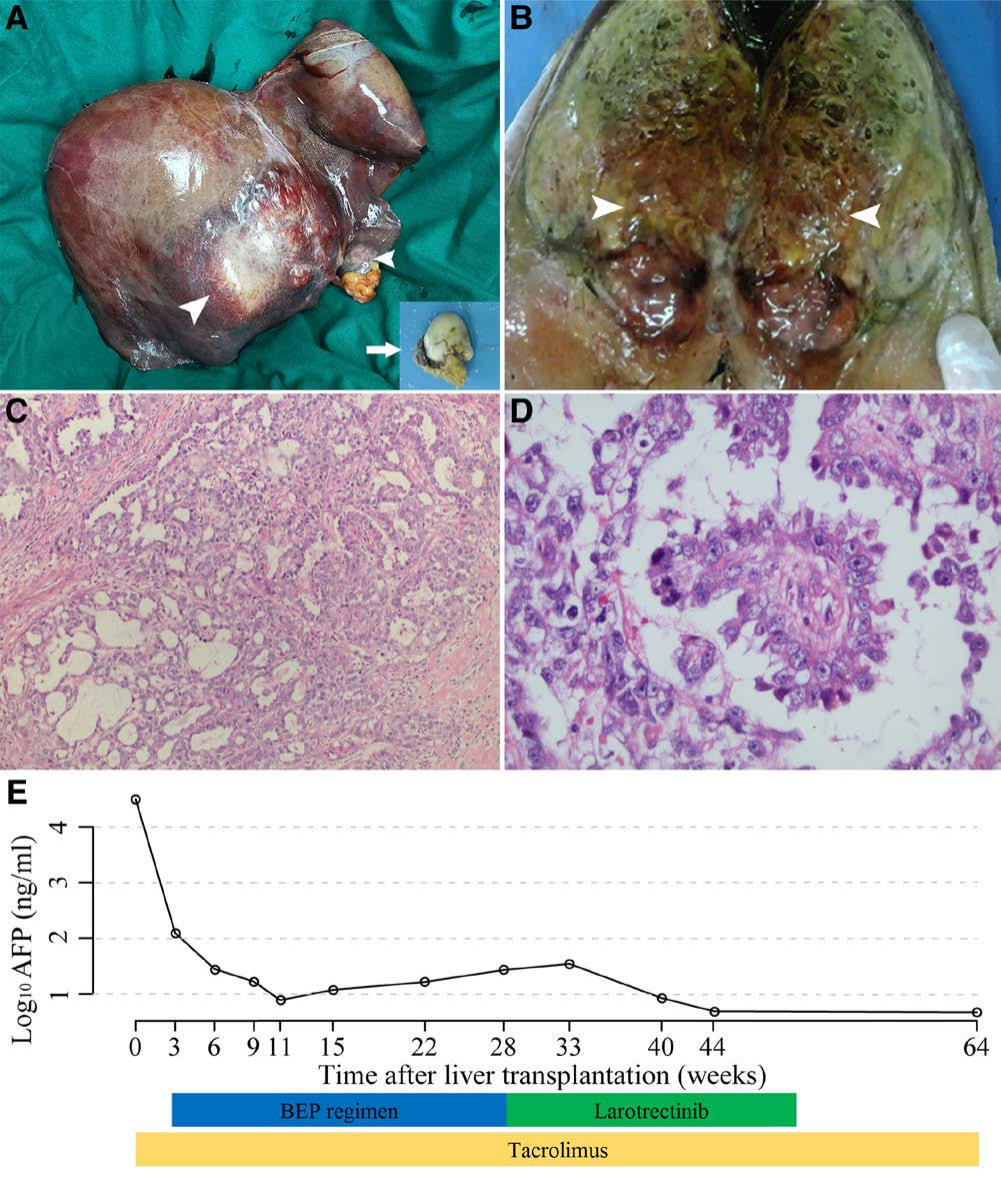
Gross specimen, histopathology, and related treatment and changes in alpha-fetoprotein (AFP) after liver transplantation. (A, B) gross specimen of the liver tumor (arrows) and intra-abdominal metastatic nodule (arrowhead); (C) histopathological features of the liver tumor in hematoxylin-eosin staining (×100); (D) Schiller-Duval bodies (×400); (E) overall process of related treatment and dynamic tendencies of AFP levels. BEP = bleomycin, etoposid, and cisplatin.

Upon multidisciplinary team consultation, TACE was performed to control liver tumor progression before addition to the active liver transplantation list (Fig. 3A and B). The patient underwent orthotopic liver transplantation and intra-abdominal nodule resection in August 2020 (Fig. 4A and B). Intraoperatively, an intra-abdominal metastatic nodule was identified in the greater omentum. Histopathological examination of the liver tumor showed typical morphological features (Fig. 4C) and Schiller-Duval bodies (central vessels surrounded by epithelial tumor cells and fibrous tissue) of YSTs (Fig. 4D). Primary hepatic YST with metastasis to the greater omentum was diagnosed.

Tacrolimus is routinely administered to prevent transplant rejections. Next-generation sequencing (NGS) of liver tumor specimens revealed *NTRK1* gene fusion and sensitivity to platinum-based chemotherapeutics. Six cycles of the adjuvant bleomycin, etoposid, and cisplatin (BEP) regimen (bleomycin 15 mg/m^2^ on day 1–3, etoposide 80 mg/m^2^ on day 1–5, and cisplatin 20 mg/m^2^ on day 1–5) were implemented postoperatively from 3 to 28 weeks. The AFP level decreased to a minimum of 7.8 ng/mL in the 11th week after liver transplantation. Thereafter, the AFP level gradually increased, reaching a maximum of 34.9 ng/mL at the 33rd week, and there was no radiological evidence of tumor collapse. The tropomyosin receptor kinase (TRK) inhibitor larotrectinib (VITRAKVI, Loxo Oncology Inc., and Bayer) was administered at a dose of 100 mg twice daily at the 28th week after the completion of the BEP regimen. The AFP level decreased to within the normal range (4.8 ng/mL) in the 44th week (Fig. 4E). Larotrectinib treatment was continued until the 50th week. The follow-up was censored on June 15, 2023. No evidence of intrahepatic recurrence or extrahepatic metastasis was found during the 34-month follow-up period.

## 3. Discussion

Primary hepatic YST are mostly sporadic. Only 15 adult cases have been reported in English literature. Primary hepatic YST is usually not considered in the differential diagnosis of liver tumors because of its rarity, and is usually misdiagnosed as HCC at presentation in adults.^[8,10,11,14,15,17]^ For malignant liver tumors with highly elevated AFP levels in adults, HCC is always the first suspected diagnosis, especially in patients with underlying chronic liver disease or liver cirrhosis. However, hepatoblastoma and primary hepatic YST are 2 important differential diagnoses that require further clarification.^[18,19]^ Hepatoblastoma occurs mostly in children aged < 5 years.^[20]^ Primary hepatic YST usually occur in young adult women without a history of chronic hepatitis or liver cirrhosis.^[8]^ Although it has been reported that cystic masses in the non-cirrhotic liver may be a radiological feature of primary hepatic YST,^[8]^ they are difficult to identify in clinical radiology procedures.

^18^F-FDG PET/CT was used to evaluate the tumor stage of the primary hepatic YST. Currently, there are no reports on ^18^F-FDG PET/CT imaging of primary hepatic YST. In this case report, in addition to the increased uptake of ^18^F-FDG in PET/CT fusion imaging, ^18^F-FDG PET/CT imaging showed no specific characteristics of a primary hepatic YST. The main advantage of ^18^F-FDG PET/CT is that a whole-body scan can initially screen out all potential lesions in the body and is helpful in determining whether the tumors in the liver are derived from primary or metastatic tumors.

The diagnosis of primary hepatic YSTs relies heavily on histopathological findings. The patient described herein did not undergo a preoperative fine-needle biopsy at presentation. According to the diagnostic criteria of the American Association for the Study of Liver Diseases, if the AFP level is highly elevated in a large tumor within a cirrhotic liver that exhibits arterial enhancement followed by venous washout on contrast-enhanced CT or magnetic resonance imaging (MRI), HCC can be identified and routine biopsy is not recommended.^[21]^ This patient had a high AFP level, and contrast-enhanced CT showed a typical pattern of arterial uptake and washout in the venous phase. Therefore, preoperative needle biopsy was not performed.

However, the preoperative diagnosis of HCC in this case is questionable. In China, the main etiology of HCC is viral hepatitis. The patient had no history of chronic liver disease or cirrhosis. Our report suggests that biopsy should be considered for liver malignancies with abnormally elevated AFP levels and without underlying liver disease, particularly in young females. It may be beneficial to preoperatively differentiate HCC, hepatoblastoma, and primary hepatic YST, as the latter 2 respond well to systemic chemotherapy.^[6,14–16,22]^

Surgical resection is the established curative treatment for primary hepatic YST. The successful removal of tumors combined with preoperative or postoperative platinum-based chemotherapy^[6,10,12,14,15]^ has been implemented in several patients with primary hepatic YST. Welan et al described a patient with a primary hepatic YST who underwent successful liver tumor resection with preoperative adjuvant chemotherapy and achieved long-term survival (>5 years).^[6]^ Hosala et al^[12]^ described a patient with primary hepatic YST who underwent successful removal of the liver tumor, followed by 4 cycles of standard adjuvant chemotherapy using the BEP regimen, with a survival time of more than 2 years. In contrast, Narita described a patient with a large primary YST who underwent partial liver tumor resection without adjuvant chemotherapy. Although the AFP level decreased to within the normal range, the patient developed a recurrent lesion, 5 cm in diameter, in the abdomen a few months after the partial hepatectomy. Following adjuvant chemotherapy with cisplatin, adriamycin, and pepleomycin, the abdominal tumor was not palpable.^[4]^

Previous studies have suggested that patients with primary hepatic YST who receive adjuvant chemotherapy have a satisfactory long-term prognosis,^[6,12]^ while those who do not receive adjuvant chemotherapy have a higher probability of tumor collapse after surgery.^[4,13]^ As primary hepatic YST are sensitive to chemotherapy, adjuvant chemotherapy remains effective, even for postoperative tumor collapse.^[4]^ Treatment protocols for primary hepatic YST similar to those for hepatoblastoma, namely, preoperative chemotherapy, surgical resection, and postoperative chemotherapy, should be considered. Due to the insufficient sample size, this hypothesis in primary hepatic YST requires further validation.

The long-term prognosis of patients with primary hepatic YST remains poor. Most patients die within 1 year due to widespread disease^[3,4,10]^ or a lack of follow-up outcomes.^[5,9,14–17]^ Limited data suggest that liver transplantation is an alternative treatment for primary hepatic YST.^[11,23]^ In this study, we report a case of multiple unresectable hepatic YSTs with extrahepatic metastasis that underwent successful liver transplantation with concurrent surgical removal of the metastasis. The patient was treated with preoperative TACE for suspected hepatocellular carcinoma (HCC). The diagnosis of primary hepatic YST was confirmed based on postoperative histopathological findings. Our study showed satisfactory long-term survival after the administration of adjuvant chemotherapy followed by the selective TRK inhibitor larotrectinib after liver transplantation. As YST has an unpredictable neoplasm biological behavior, close clinical follow-up will be continued to validate our treatment protocols.

## 4. Conclusion

For young adults with abnormally elevated serum AFP levels in a non-cirrhotic liver, primary hepatic YST should be considered as an alternative diagnosis of HCC. Fine-needle biopsy is recommended in highly suspected cases of primary hepatic YST because YST is a chemotherapy-sensitive tumor that can benefit from adjuvant chemotherapy. Our research suggests that multimodal therapy dominated by liver transplantation, including preoperative TACE, postoperative adjuvant chemotherapy, and TRK inhibitors, is feasible for unresectable primary hepatic YST.

## Author contributions

**Conceptualization:** Guang-Hua Liu, Ming-Ke Qiu, Yang Wang, Ting-Ting Zhang, Li-Jun Wang, Wen-Bin Guan, Jing-Min Ou, Li-Tian Chen.

**Data curation:** Li-Tian Chen.

**Formal analysis:** Guang-Hua Liu.

**Funding acquisition:** Ming-Ke Qiu, Jing-Min Ou.

**Software:** Guang-Hua Liu, Yang Wang, Ting-Ting Zhang, Li-Jun Wang, Wen-Bin Guan.

**Supervision:** Jing-Min Ou.

**Visualization:** Wen-Bin Guan.

**Writing – original draft:** Guang-Hua Liu, Ming-Ke Qiu, Yang Wang, Ting-Ting Zhang, Li-Jun Wang, Li-Tian Chen.

**Writing – review & editing:** Jing-Min Ou, Li-Tian Chen.
